# Proteomic Analysis of Protein Ubiquitination Events in Human Primary and Metastatic Colon Adenocarcinoma Tissues

**DOI:** 10.3389/fonc.2020.01684

**Published:** 2020-09-10

**Authors:** Yi Zhang, Cong Chen, Tingting Yu, Tao Chen

**Affiliations:** ^1^Department of Colorectal Surgery, The First Affiliated Hospital of Nanjing Medical University, Nanjing, China; ^2^Department of Neurology, The First Affiliated Hospital of Nanjing Medical University, Nanjing, China; ^3^Department of Medical Genetics, School of Basic Medical Science, Jiangsu Key Laboratory of Xenotransplantation, Nanjing Medical University, Nanjing, China

**Keywords:** colon adenocarcinoma, proteomics, ubiquitination, metastasis, therapeutic targets

## Abstract

Protein ubiquitination is essential for multiple physiological processes through regulating the stability or function of target proteins and has been found to play critical roles in human cancers. However, the protein ubiquitination profile of human metastatic colon adenocarcinoma tissue has not been elucidated yet. In this study, a proprietary ubiquitin branch (K-ε-GG) antibody-based label-free quantitative proteomics and bioinformatics were performed to identify the global protein ubiquitination profile between human primary (Colon) and metastatic colon adenocarcinoma (Meta) tissues. A total of 375 ubiquitination sites from 341 proteins were identified as differentially modificated (| Fold change| > 1.5, *p* < 0.05) in Meta group compared with the Colon group. Among them, 132 ubiquitination sites from 127 proteins were upregulated and 243 ubiquitination sites from 214 proteins were downregulated in Meta group. Fifteen ubiquitination motifs were found. Furthermore, GO and KEGG pathway analysis indicated that proteins with altered ubiquitination in Meta group were enriched in pathways highly related to cancer metastasis, such as RNA transport and cell cycle. We speculate that the altered ubiquitination of CDK1 may be a pro-metastatic factor in colon adenocarcinoma. This study provides novel scientific evidences to elucidate the biological functions of protein ubiquitination in human colon adenocarcinoma and insights into its potential mechanisms of colon cancer metastasis, which would be helpful to discover novel biomarkers and therapeutic targets for effective treatment of colon cancer.

## Introduction

Colorectal cancer is one of the most common and lethal cancer worldwide with about 70% of cases arise in the colon (1). More than 1 million individuals develop colorectal cancer annually (2) and adenocarcinomas make up over 95% of colorectal cancers (3). Unfortunately, nearly 20% of patients with primary colorectal cancer encounter distant metastasis at the time of diagnosis. Moreover, only 10–30% of patients with distant metastasis can have potentially curative resection of the primary tumor and the distant metastasis (4). The disease-related mortality corresponds to approximately 33% in the developed world and the 5-year survival rates is only 10% for distant metastatic cases (2). Although great developments have been made in the diagnosis and treatment for colorectal cancer, the overall survival rate of the patients rarely improved (5). The post-operative recurrence (6) and metastasis (7) remain the two obstacles for colorectal cancer therapy. Thus, it is necessary to elucidate the metastatic mechanisms of colorectal cancer to discover novel biomarkers and therapeutic strategies for effective diagnosis and treatment.

Ubiquitination is an important post-translational modification that has a major role in the modulation or degradation of cellular proteins. The process involves three enzymes: ubiquitin-activating enzyme E1, ubiquitin-coupled enzyme E2, and ubiquitin ligase E3, transferring ubiquitin to an internal lysine (K) residue on the protein substrate, which can lead to protein mono-ubiquitination or poly-ubiquitination (8). Protein ubiquitination regulates a wide variety of biological processes such as DNA repair, cell cycle regulation, signal transduction, apoptosis, and oncogenesis/metastasis (9, 10). Ubiquitination is a reversible process in which de-ubiquitinizing enzymes can catalyze de-ubiquitination preventing degradation of proteins (11). It is worth noting that poly-ubiquitination by linkage of ubiquitin at K48 regulates protein stability via targeting substrate proteins for 26S proteasome degradation. However, poly-ubiquitination through ubiquitin K63 regulates signal transduction, kinase activation and endocytosis (12). In addition, mono-ubiquitination acts more like a signaling marker that controls various processes such as membrane transport and transcription (13). Accumulating evidence indicates that cancer cells could modulate the members of the ubiquitination pathway to stabilize aberrant oncogenic signaling. For example, Ubc13, an E2 enzyme that catalyzes K63-linked protein polyubiquitination, is required for breast cancer metastasis *in vivo* through increasing the metastasis-associated gene expression (14). Recently, proteomic analysis of ubiquitinated proteins has become an effective method to globally identify and quantify protein ubiquination in cancer cells (15) and tissues (8). However, the ubiquitinated protein profile of human primary and metastatic colon adenocarcinoma tissues remains to be elucidated.

Proteomic analysis of ubiquitination has greatly improved with the commercialization of antibodies specific for the diglycine remnant left on ubiquitinated lysine residues (K-ε-GG) after trypsin digestion of ubiquitin-modified proteins (16). In this study, we identified the ubiquitinated protein profile of human primary and metastatic colon adenocarcinoma tissues using anti-ubiquitin remnant motif antibody (specific to K-ε-GG)-based affinity enrichment combined with LC-MS/MS analysis. Then, GO enrichment, KEGG pathway, protein-protein interaction network and motif analysis were used to investigate possible mechanism that the altered protein ubiquitination involved in. Our preliminary study revealed the global profiling of ubiquitinated proteins in colon adenocarcinoma and will help to elucidate the molecular mechanisms associated with metastasis to discover better biomarkers and therapeutic targets for colorectal cancer.

## Materials and Methods

### Samples

Metastatic colon adenocarcinoma tissues (Meta, *n* = 3, pathologic stage: pT3N1Mx) were obtained from the First Affiliated Hospital of Nanjing Medical University, China, as approved by the Ethics Committee of the First Affiliated Hospital of Nanjing Medical University. Age matched primary colon adenocarcinoma tissues (Colon, *n* = 3, pathologic stage: pT3N0Mx) were obtained as control. Written informed consent was obtained from each patient after full explanation of the purpose and nature of all experimental procedures. All the tissues were collected during surgery and immediately frozen in liquid nitrogen and stored at −80°C until use.

### Protein Extraction

The proteins were extracted as previously described (17) with minor modifications. Briefly, each tissue sample was individually incubated in lysis buffer (8 M Urea, 10 mM EDTA, 10 mM DTT, 1% Protease Inhibitor Cocktail), followed by sonication three times on ice using a high intensity ultrasonic processor. The remaining debris was removed by centrifugation at 12,000 r/min at 4°C for 10 min. Finally, the supernatant was collected and the protein concentration was determined with 2D Quant kit according to the manufacturer’s instructions.

### Trypsin Digestion and HPLC Fractionation

For digestion, the protein solution was reduced with 10 mM DTT for 1 h at 56°C and alkylated with 30 mM iodoacetamide for 45 min at room temperature in darkness. The protein sample was then diluted by adding 100 mM NH_4_HCO_3_ to urea concentration less than 2 M. Finally, trypsin was added at 1:50 trypsin-to-protein mass ratio for the first digestion overnight and 1:100 trypsin-to-protein mass ratio for a second 4 h-digestion. Peptides were loaded on Strata–X C18 pillar for three times, washed with 0.1% FA + 5% ACN twice, and eluted with 1 ml 0.1% FA + 80% ACN. Eluted peptides were dried with Vacuum concentration meter.

The tryptic peptides were fractionated into fractions by high pH reverse-phase HPLC using Shimadzu LC20AD C18 column (5 μm particles, 10 mm ID, 250 mm length). The peptides were combined into four fractions and dried by vacuum centrifuging.

### Affinity Enrichment

To enrich Kub *modified* peptides, tryptic peptides dissolved in NETN buffer (100 mM NaCl, 1 mM EDTA, 50 mM Tris–HCl, 0.5% NP-40, pH 8.0) were incubated with anti-Lys-ε-Gly-Gly (K-ε-GG) remnant antibody beads (PTMScan ubiquitin remnant motif K-ε-GG kit, Cell Signaling Technology) at 4°C overnight with gentle shaking. Then the beads were washed four times with NETN buffer and twice with H_2_O. The bound peptides were eluted from the beads with 0.1% trifluoroacetic acid. Finally, the eluted fractions were combined and vacuum-dried. For LC-MS/MS analysis, the resulting peptides were desalted with C18 ZipTips (Millipore) according to the manufacturer’s instructions.

### LC-MS/MS Analysis

Each fraction was resuspended in buffer A (0.1%FA) and centrifuged at 20,000 r/min for 10 min. The supernatant was loaded on Thermo Scientific^TM^ UltiMate^TM^ 3000 UHPLC system equipped with a trap and an analytical column. The samples were loaded on a trap column at 5 μL/min for 8 min, and then eluted into the homemade nanocapillary C18 column (ID 75 μm × 25 cm, 3 μm particles) at a flow rate 250 nl/min. The gradient of buffer B (98%ACN, 0.1%FA) was increased from 5 to 25% in 40 min, and then increased to 35% in 5 min, followed by 2 min linear gradient to 80%, then maintenance at 80% B for 2 min, and finally return to 5% in 1 min and equilibrated for 6 min.

The peptides separated by liquid phase chromatography were ionized by a nanoESI source and then passed to a tandem mass spectrometer Q-Exactive HF X (Thermo Fisher Scientific, San Jose, CA, United States) for DDA (data-dependent acquisition) mode detection. The main parameters were set: ion source voltage was set to 2 kV, MS1 scanning range was 350–1800 m/z; resolution was set to 60,000; MS2 starting m/z was fixed at 100; resolution was 30,000. The ion screening conditions for MS2 fragmentation: charge 2+ to 6+, and the top 15 parent ions with the peak intensity exceeding 20,000. The ion fragmentation mode was HCD, and the fragment ions were detected in Orbitrap. The dynamic exclusion time was set to 30 s. The AGC was set to: MS1 3E6, MS2 1E5.

### Database Search

The resulting MS/MS data were processed using Maxquant search engine (v1.5.2.8). Tandem mass spectra were searched against SwissProt Human database concatenated with reverse decoy database. Trypsin/P was specified as cleavage enzyme allowing up to two missing cleavages. The mass tolerance for precursor ions was set as 20 ppm in First search and 5 ppm in Main search, and the mass tolerance for fragment ions was set as 0.05 Da. Carbamidomethyl on Cys was specified as fixed modification and Gly-Gly modification for lysines and oxidation on Met were specified as variable modifications. Label-free quantification method was LFQ, FDR was adjusted to <1%.

### Western Blotting

Three metastatic colon adenocarcinoma tissues (pathologic stage: pT3N1Mx) were mixed as the Meta (M) protein sample, and three age matched primary colon adenocarcinoma tissues (pathologic stage: pT3N0Mx) were mixed as Colon (C) protein sample. The equal-load amount of C and M samples were used for Western blotting analysis. Anti-CDK1 antibody (Abcam, United States) was used to detect the protein level of CDK1. Signals were detected with Immobilon ECL reagents (Millipore, United States).

### Bioinformatics and Statistical Analysis

Differentially ubiquitinated proteins of metastatic colon adenocarcinoma (Meta) tissues compared with primary colon adenocarcinoma tissues (Colon) were determined by the significance criteria (A 1.5-fold cut-off was used to determine quantitative changes of up-regulated and down-regulated ubiquitinated proteins, with a *p* < 0.05). Gene ontology (GO) annotations and KEGG pathway on the proteomics level are derived from the David database. The clustering relationship is visualized using the heat map drawn by the function heatmap.2 in the R language package gplots. A volcano map was prepared by using negative log-log10 (*p*-value) as the ordinate (using | FC| ≥ 1.5, significant *p*-value ≤ 0.05 to screen differentially expressed proteins). Principal component analysis (PCA) analysis was conducted using the prcomp command of the R statistical software. Motif analysis software^1^ was used to analyze the model of ubiquitinated peptide sequences in specific positions of modify-21-mers (10 amino acids upstream and downstream of the site) in all protein sequences. And all the database protein sequences were used as background database parameter, other parameters with default. String website and Cytospace software were employed to analyze the interaction and the potential function of the ubiquitinated proteins. The results of bioinformatics analysis were visualized using GraphPad Prism 7 software.

## Results

### Identification of the Ubiquitin-Modified Proteome in Human Primary and Metastatic Colon Adenocarcinoma Tissues

**FIGURE 1:**
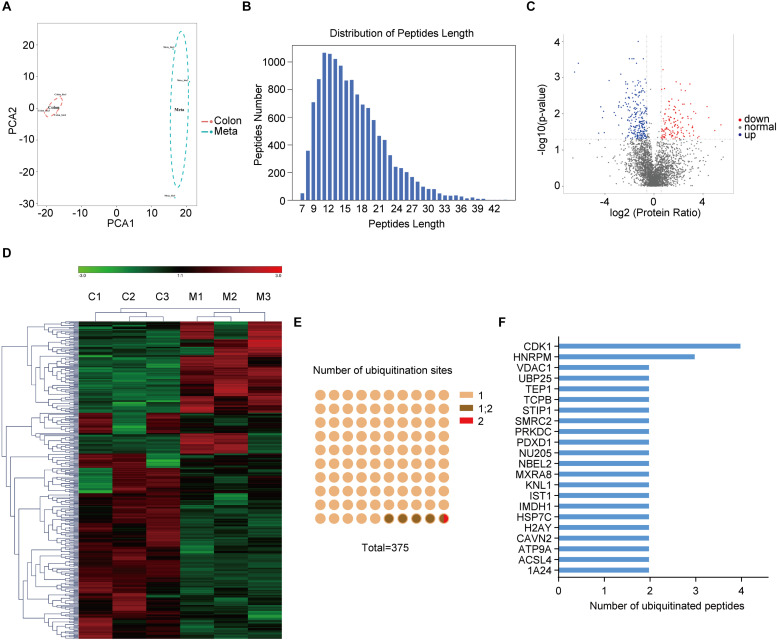
The ubiquitination profile in human primary and metastatic colon adenocarcinoma tissues. **(A)** Principal component analysis (PCA) shows the statistical discrimination between Mass Spectrometry (MS) profile of the primary (Colon) and metastatic (Meta) colon adenocarcinoma tissues. The highlighted area for each component corresponds to a confidence interval of 95%. **(B)** Distribution of peptide length of all ubiquitinated peptides. **(C)** Volcano plot showing ubiquitylated proteins that are significantly (| Fold change| > 1.5, *p* < 0.05) different between the Meta and Colon group. **(D)** Heat map illustrating identified proteins with different ubiquitin modification in Meta (M) and Colon (C) group. **(E)** Number of ubiquitination sites on the identified ubiquitinated peptides. **(F)** Ubiquitinated peptides shared the same parent proteins.

Anti-K-ε-GG antibody enrichment-based label-free quantification of whole proteome was used to investigate the differential ubiquitylation in metastatic colon adenocarcinoma tissues (Meta) and the primary ones (Colon). PCA revealed significant differences between subtypes within the first principal component, clearly separating Meta group from Colon group (Figure 1A). Peptides derived from ubiquitylated proteins always include a di-glycine remnant on modified lysine residues (18), indicating the prior conjugation of ubiquitin to that region of the parent protein. Thus, ubiquitin remnant-containing peptides have been used to identify ubiquitination sites (19). In sum, a total of 12,886 ubiquitination sites on 4138 proteins was identified, of which 6,990 sites on 2880 proteins have quantitative information. Most ubiquitinated peptides identified in this study were less than 24 amino acids in length (Figure 1B). 375 ubiquitination sites from 341 proteins were identified as differentially modificated (| Fold change| > 1.5, *p* < 0.05) in metastatic colon adenocarcinoma tissues (Meta) compared with the primary ones (Colon). Among them, 132 ubiquitination sites from 127 proteins were upregulated and 243 ubiquitination sites from 214 proteins were downregulated in Meta group (Figure 1C). The representative differential ubiquitinated proteins (| Fold change| > 2, *p* < 0.05) were listed in Supplementary Table 1. The results of hierarchical clustering showed the greatest difference of ubiquitylated proteins between the Meta and Colon groups (Figure 1D). Among ubiquitinated peptides, 95.2% peptides contained only one ubiquitinated site, 4.53% peptides contained one or two ubiquitination sites, and 0.27% peptides contained two ubiquitination sites (Figure 1E). Many ubiquitinated peptides derived from same parent protein, and the largest number of ubiquitinated peptides was derived from CDK1 (Figure 1F).

### Functional Enrichment and Protein Interaction Networks of Altered Ubiquitinated Proteins in Metastatic Colon Adenocarcinoma

**FIGURE 2:**
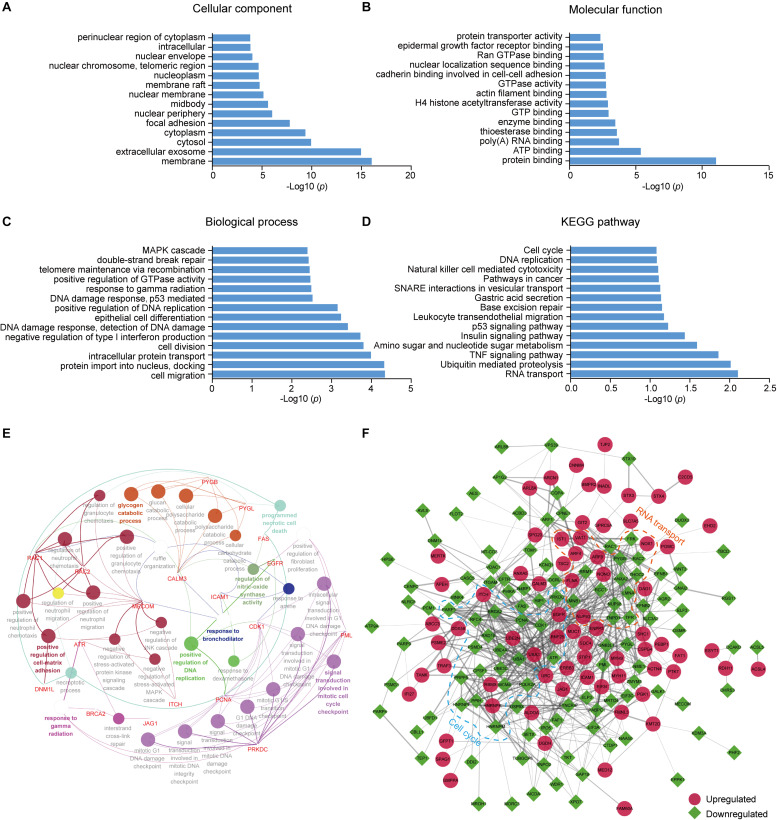
GO and KEGG pathway enrichment analysis and functional protein association networks of altered ubiquitinated proteins. **(A)** Cellular component. **(B)** Molecular function. **(C)** Biological process. **(D)** KEGG pathways. **(E)** Functionally grouped network of enriched categories was generated using ClueGO. GO terms are represented as nodes and functionally grouped networks are linked to their biological function, where the most significant term in the group is labeled. Visualization has been carried out using Cytoscape software. **(F)** The protein–protein interaction network of the differentially modificated proteins was constructed using Cytoscape software. The upregulated proteins are represented as red circle while the downregulated proteins are represented as green square. Proteins in significant networks were annotated and grouped with simplified GO terms surrounded by dashed lines.

To better understand the lysine ubiquitome changes in metastatic colon adenocarcinoma, we performed GO and pathway functional enrichment analysis. In the cellular compartment category, proteins with altered ubiquitination were associated with membrane, extracellular exosome, cytosol, cytoplasm, focal adhesion, and nuclear periphery, etc. (Figure 2A). In the molecular function category, the altered ubiquitinated proteins were associated with protein binding, ATP binding, poly (A) RNA binding, enzyme binding, GTPase activity, H4 histone acetyltransferase activity, etc. (Figure 2B). In the biological process category, the altered ubiquitinated proteins were involved in cell migration, intracellular protein transport, cell division, negative regulation of type I interferon production, DNA damage response, epithelial cell differentiation, telomere maintenance via recombination, MAPK cascade, etc. (Figure 2C). The result of KEGG pathway analysis indicated that RNA transport, ubiquitin mediated proteolysis, TNF signaling pathway, amino sugar and nucleotide sugar metabolism, insulin signaling pathway, p53 signaling pathway, base excision repair, and cell cycle, etc. were the most prominent pathways enriched in proteins with altered ubiquitination (Figure 2D). ClueGO analysis was applied to identify interpretation and interrelations of enriched categories in functionally grouped network. We observed significant enrichment for signal transduction involved in mitotic cell cycle checkpoint, positive regulation of cell-matrix adhesion, positive regulation of DNA replication, regulation of nitric-oxide synthase activity, programmed necrotic cell death, glycogen catabolic process (Figure 2E), all of which are associated with cancer.

To further elucidate the significance of ubiquitination in metastatic colon adenocarcinoma, we generated an interaction network using string database. Two sub-networks including RNA transport and cell cycle were identified. In the sub-network of RNA transport, some proteins showed increased ubiquitination, while others showed decreased ubiquitination. Interestingly, the components in the sub-network of cell cycle had greatly decreased ubiquitination levels. These proteins included CDK1, PCNA, MCM4, PRKDC, and ATR (Figure 2F).

### Motif Analysis of Altered Ubiquitinated Proteins

**FIGURE 3:**
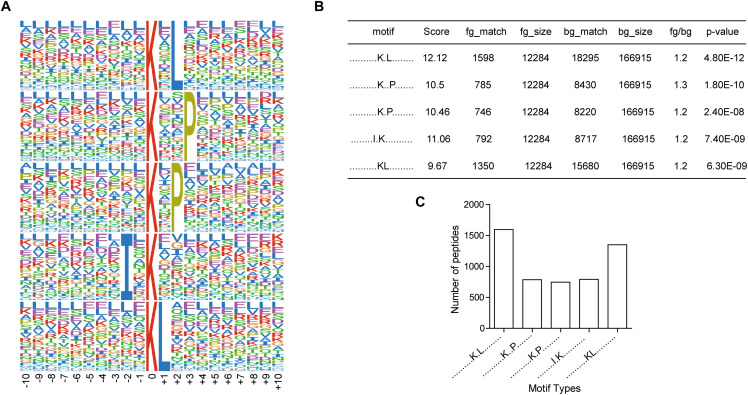
Motif analysis of all the altered ubiquitination sites in Meta group. **(A)** Five representative ubiquitination motifs. The height of each letter corresponds to the frequency of this amino acid residue in its position. The central K refers to the ubiquitinated Lys. **(B)** The parameters of five representative ubiquitination motifs. **(C)** Number of identified peptides containing ubiquitinated K in each motif.

To investigate the regulation of ubiquitination in human metastatic colon adenocarcinoma, ubiquitination motif analysis was carried out using Motif-X software. Five significantly distinguished motifs were shown in Figure 3A, including K^∗^-X(1)-L, K^∗^-X(2)-P, K^∗^-X(1)-P, I-X(1)-K^∗^, and K^∗^L, which refers to 1598, 785, 746, 792, and 1350 unique ubiquitinated peptides (Figures 3B,C), respectively (K^∗^: the ubiquitinated lysine residue; X: any amino acid residue). All 15 identified motifs were listed in Table 1.

**TABLE 1:** The identified ubiquitination motifs.

regexp	Score	fg_match	fg_size	bg_match	bg_size	fg/bg	*p*-value
.K.L.	12.12	1598	12284	18295	166915	1.2	4.80E-12
.K.P.	10.5	785	12284	8430	166915	1.3	1.80E-10
.K.P.	10.46	746	12284	8220	166915	1.2	2.40E-08
.I.K.	11.06	792	12284	8717	166915	1.2	7.40E-09
.KL.	9.67	1350	12284	15680	166915	1.2	6.30E-09
.KQ.	9.55	729	12284	7647	166915	1.3	2.10E-11
.KG.	9.71	841	12284	9101	166915	1.3	1.50E-10
.AK.	9.84	950	12284	10342	166915	1.2	2.80E-11
.L.K.	8.67	1548	12284	18113	166915	1.2	2.20E-09
.F.K.	6.73	549	12284	6367	166915	1.2	2.00E-04
.A.K.	7.07	930	12284	10976	166915	1.2	1.50E-05
.L.K.	6.72	1394	12284	16010	166915	1.2	2.90E-10
.KI.	6.47	694	12284	8291	166915	1.1	5.40E-04
.LK.	7.29	1432	12284	17348	166915	1.1	7.20E-06
.KV.	6.08	890	12284	10735	166915	1.1	2.70E-04

*fg: foreground; bg: background.*

### Further Analysis of Metastasis-Associated Protein Ubiquitination in Colon Adenocarcinoma

**FIGURE 4:**
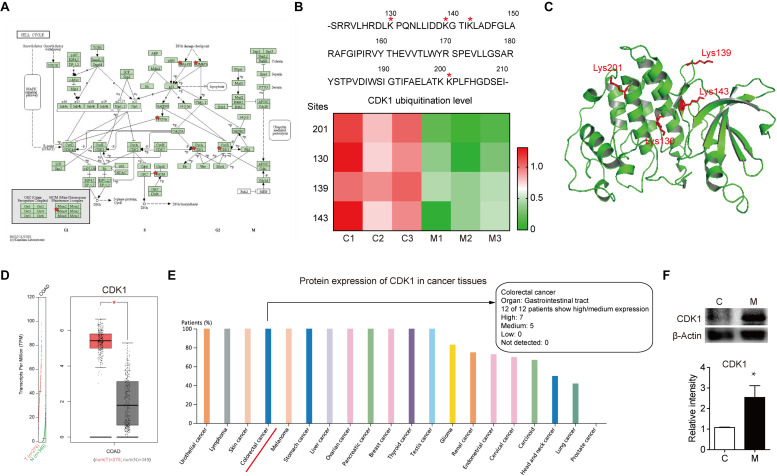
The altered ubiquitination of CDK1 in colon adenocarcinoma is highly associated with metastasis. **(A)** The Cell cycle KEGG pathway which was achieved by DAVID pathway analysis. The altered ubiquitinated proteins in Meta group are labeled with red stars. **(B)** The four identified ubiquitination sites of CDK1 are marked with red stars. The abundance of four ubiquitinated peptides in Colon (C) and Meta (M) group is visualized using the heat map. **(C)** The structure of CDK1 (PDB ID: 4Y72) is used as a reference. The four identified ubiquitination sites are shown as sticks in red. **(D)** The gene expression profile of CDK1 in colon adenocarcinoma samples and paired normal tissues. | Log_2_FC| cutoff: 1; *q* value cutoff: 0.01. COAD: colon adenocarcinoma. **(E)** The protein expression profile of CDK1 across all tumor samples was plotted using Proteinatlas database. CDK1 protein expression in colorectal cancer is labeled with red line. **(F)** Representative western blotting and quantification data of protein CDK1 in Colon (C) and Meta (M) group. *p < 0.05.

After comprehensive analysis of ubiquitination data, GO enrichment and KEGG pathways, the cell cycle signaling pathway was chosen for further analysis. As shown in Figure 4A, there are six identified proteins (CDK1, PCNA, MCM4, PRKDC, and ATR) involved in the cell cycle. In particular, the ubiquitination level of these six proteins was all significantly decreased in metastatic colon adenocarcinoma, indicating the important role of cell cycle related protein ubiquitination in tumor metastasis. Since the largest number of altered ubiquitinated peptides was derived from CDK1 (Figure 1F), the ubiquitination of CDK1 was further analyzed. We found four ubiquitinated peptides with four ubiquitination sites (130, 139, 143, and 201) in CDK1 and the abundance of all these four ubiquitinated peptides was significantly reduced in Meta group compared with Colon group (Figure 4B). The four ubiquitination sites in the 3D structure of CDK1 was shown in Figure 4C. Data extracted from TCGA database revealed that CDK1 expression was notably higher in colon adenocarcinoma samples compared to matched TCGA normal tissues and GTEx data (Figure 4D). Moreover, the protein expression level of CDK1 in 7 out of 12 patients with colorectal cancer was high (Figure 4E). We further determined the protein level of CDK1 using Western blotting. The result showed that protein CDK1 was significantly upregulated in metastatic colon adenocarcinoma tissue (Figure 4F). Therefore, the decreased ubiquitination level of CDK1 might explain the high protein level in colon adenocarcinoma, which is highly associated with tumor metastasis.

## Discussion

Ubiquitination regulates diverse aspects of protein function, including degradation, protein transportation and protein-protein interaction (13). Dysregulation of ubiquitination has been implicated in many human diseases, including cancers (20). It has been demonstrated that protein ubiquitination controls the expression level of some key oncoproteins and tumor suppressors that involved in cancer development. For example, E3 ligases such as WD repeat domain-containing7 (FBW7) (21) and the anaphase promoting complex/cyclosome (APC/C) (22) function as tumor suppressors by downregulating oncoproteins. In contrast, Skp2 (23) and MDM2 (24) ubiquitinate and inhibit tumor suppressors through proteasomal degradation serving as oncogenic factors. Therefore, targeting the ubiquitin-proteasome system may be effective for certain cancer treatment (25). As we know, overexpression and dysregulation of β-catenin/Wnt signaling pathway is involved in colon cancers. Many inhibitors that interrupt β-catenin/Wnt signaling have been studied as candidates for colon cancer treatment (26). Recent evidence demonstrated that neutral red (NR) suppressed proliferation and migration of colon cancer cells by regulating β-catenin/Wnt Signaling. NR could promote the ubiquitination and proteasomal degradation of endogenous β-catenin via impairing the deubiquitinating activity of ubiquitin-specific protease 4 (USP4) in colon cancer cells (20). In addition, an E3 ubiquitin ligase, tripartite motif-containing protein 65 (TRIM65), has been found to modulate the migration and metastasis of colorectal cancer cells. Thus, it is rationale to presume that the global profiling of ubiquitinated proteins in colon cancer is different, especially in metastatic colon adenocarcinoma.

The results of MS data showed that the global protein ubiquitination in human metastatic colon adenocarcinoma is notably different from primary colon adenocarcinoma. The KEGG pathway analysis indicated that RNA transport, ubiquitin mediated proteolysis, TNF signaling pathway, amino sugar and nucleotide sugar metabolism, insulin signaling pathway, p53 signaling pathway, base excision repair, and cell cycle, etc. were the most prominent pathways enriched in proteins with altered ubiquitination, which are associated with tumor metastasis. For example, Guo et al. found that hTREX84 that links transcription elongation to mRNA transport was culprit of aggressive human breast cancers (27). Boram et al. revealed that in lung and colon carcinoma cells, TNF signaling enhance cancer cell survival and liver metastasis by upregulating IL-6 expression via IGF-I receptor (28). Hyperglycemia has been reported to induce tumor growth, invasion and metastasis (29). Lu et al. demonstrated that insulin triggers cell proliferation and metastatic effects on human colorectal cancer cells which is regulated by insulin receptor signaling and the PI3K/Akt pathway (30). Loss of p53 not only induce tumor initiation and progression but also allows tumors to more quickly gain a full repertoire of metastatic facilitators (31). Modulation of base excision repair can alter cell metabolism and response to DNA damage and thereby overcome 5-FU chemotherapeutic resistance in colorectal cancer (32). Deregulation of the cyclin-dependent kinase subunits (CDKs) in cell cycle always leads to an uncontrolled proliferation of cancer cells and hence cell cycle is a crucial therapeutic target for anti-cancer treatment (33).

In the present study, we propose CDK1 as the pro-metastatic protein in colon adenocarcinoma. Firstly, we identified the largest number of altered ubiquitinated peptides derived from CDK1 in metastatic colon adenocarcinoma tissues. Secondly, cell cycle was one of the most prominent pathways enriched in proteins with altered ubiquitination. Thirdly, most of the components in the cell cycle sub-network had greatly decreased ubiquitination levels. Fourthly, we found four ubiquitinated peptides with four ubiquitination sites (130, 139, 143, and 201) in CDK1 and the abundance of all these four ubiquitinated peptides was significantly reduced in Meta group compared with Colon group. Finally, the protein expression level of CDK1 is generally high in colorectal cancer. CDK1 plays an important role in the regulation of cell cycle, including the G2/M transition, G1 progression and G1/S transition. The protein level of CDK1 is held at a constant steady level throughout the cell cycle process, which is maintained by a coordinated regulation of protein synthesis and ubiquitin-proteosomal degradation (34). CDK1 can be ubiquitinated by the E3 ubiquitin ligase SCFβTrCP and degraded by the lysosome. Furthermore, CDK1 accumulation was positively correlated to the degree of tumor malignancy and showed a negative correlation with βTrCP (34). CDK1 overexpression has been documented in lung cancer, lymphoma, and advanced melanoma (35). A high CDK1 nuclear/cytoplasmic ratio was correlated with poor overall survival of colorectal cancer patients (36). In our view, the altered ubiquitination of CDK1 may explain its increased protein level and abnormal subcellular localization in human colorectal cancer. Indeed, we found the protein level of CDK1 was significantly evaluated in in metastatic colon adenocarcinoma tissue. Our result suggests that the decreased ubiquitination of CDK1 may be a potential mechanism of human colon cancer metastasis. It is important to note that the anti-K-ε-GG antibody also enriches for neddylation and ISGylation, although the abundance is very low (37). Therefore, the K-ε-GG enrichment method can be combined with stable isotope (SILAC) amino acids labeled samples to achieve relative quantification of protein ubiquitination in the future study (16). Due to the limitations of this current study, further studies that enroll larger cancer samples and elucidate the underlying mechanisms of these altered ubiquitinated proteins are of great importance to prove our presumption.

## Conclusion

In conclusion, this is the first report on the differential protein ubiquitination profile of human primary and metastatic colon adenocarcinoma tissues. This study offers new insights to understand ubiquitination-mediated multiple cellular biological processes to discover better biomarkers and therapeutic targets for colorectal cancer. This study would serve as a valuable reference for biological functions of protein ubiquitination in human metastatic colon cancer.

## Data Availability Statement

The datasets presented in this study can be found in online repositories. The names of the repository/repositories and accession number(s) can be found below: ProteomeXchange, PXD020597 (http://proteomecentral.proteomexchange.org/cgi/GetDataset?ID=PXD020597).

## Ethics Statement

The studies involving human participants were reviewed and approved by the Ethics Committee of the First Affiliated Hospital of Nanjing Medical University. The patients/participants provided their written informed consent to participate in this study.

## Author Contributions

YZ and CC collected and analyzed the data. TY and TC designed the study and wrote the manuscript. All authors contributed to the article and approved the submitted version.

## Conflict of Interest

The authors declare that the research was conducted in the absence of any commercial or financial relationships that could be construed as a potential conflict of interest.

## Footnotes

1 http://meme-suite.org/
