# Supplementary material for: Proteomic Analysis of Protein Ubiquitination Events in Human Primary and Metastatic Colon Adenocarcinoma Tissues
Source: Front Oncol. 2020 Sep 10;10:1684. doi: 10.3389/fonc.2020.01684 (PMC7511592; doi:10.3389/fonc.2020.01684)
Supplement: Supplementary file 1 [file Table_1.DOCX]

**Table 1. Ubiquitinated proteins identified in metastatic colon adenocarcinoma relative to primary colon adenocarcinoma**

| **Protein ID** | **Gene name** | **Protein name** | **Peptide Sequence** | **Peptide length** | **Number of GlyGly (K)** | **Position in peptide** | **Position in protein** | **Site PEP** | **Site Score** | **Meta/Colon** | **Meta/Colon P-value** | **Diff_state** |
| --- | --- | --- | --- | --- | --- | --- | --- | --- | --- | --- | --- | --- |
| O75110 | ATP9A | Probable phospholipid-transporting ATPase IIA | TVWLGHPEKR | 10 | 1 | 9 | 48 | 2.43E-05 | 94.662 | 44.078 | 0.0202 | up |
| P31431 | SDC4 | Syndecan-4 | DEGSYDLGKKPIYK | 14 | 1 | 9 | 184 | 1.30E-19 | 115.71 | 31.211 | 0.029 | up |
| Q9C0K1 | S39A8 | Zinc transporter ZIP8 | TYGQNGHTHFGNDNFGPQEKTHQPK | 25 | 1 | 20 | 241 | 1.53E-45 | 96.718 | 22.206 | 0.0128 | up |
| Q8N0X7 | SPART | Spartin | TRPSSDQLKEASGTDVK | 17 | 1 | 9 | 362 | 1.77E-69 | 136.17 | 20.129 | 0.0063 | up |
| P49815 | TSC2 | Tuberin | SNVLLSFDDTPEKDSFR | 17 | 1 | 13 | 930 | 9.33E-10 | 71.692 | 12.726 | 0.042 | up |
| P21333 | FLNA | Filamin-A | VKCSGPGLSPGMVR | 14 | 1 | 2 | 1452 | 1.43E-05 | 75.695 | 11.83 | 0.0461 | up |
| Q9NYT0 | PLEK2 | Pleckstrin-2 | IVDKMHDSNTGIR | 13 | 1 | 4 | 135 | 1.43E-145 | 174.41 | 10.592 | 0.0097 | up |
| O75410 | TACC1 | Transforming acidic coiled-coil-containing protein 1 | KCAQDYLAR | 9 | 1 | 1 | 714 | 7.89E-10 | 126.67 | 9.4636 | 0.0087 | up |
| Q96J02 | ITCH | E3 ubiquitin-protein ligase Itchy homolog | VYYVDHVEKR | 10 | 1 | 9 | 350 | 1.12E-06 | 107.57 | 9.3761 | 0.0297 | up |
| Q13049 | TRI32 | E3 ubiquitin-protein ligase TRIM32 | KGFLKEIR | 8 | 1 | 5 | 405 | 0.0304024 | 70.525 | 9.3331 | 0.039 | up |
| P57739 | CLD2 | Claudin-2 | VKSEFNSYSLTGYV | 14 | 1 | 2 | 218 | 4.69E-23 | 118.18 | 8.8679 | 0.024 | up |
| Q9UHP3 | UBP25 | Ubiquitin carboxyl-terminal hydrolase 25 | YLSYGSGPKR | 10 | 1 | 9 | 444 | 0.0011211 | 62.463 | 8.8663 | 0.0219 | up |
| Q8IWZ3 | ANKH1 | Ankyrin repeat and KH domain-containing protein 1 | LNKNVPTNVR | 10 | 1 | 3 | 1833 | 1.78E-05 | 78.939 | 8.6933 | 0.024 | up |
| P38159 | RBMX | RNA-binding motif protein, X chromosome | LFIGGLNTETNEKALEAVFGK | 21 | 1 | 13 | 22 | 2.03E-76 | 129.32 | 8.1744 | 0.0137 | up |
| O60488 | ACSL4 | Long-chain-fatty-acid--CoA ligase 4 | NHYLKDIER | 9 | 1 | 5 | 702 | 1.42E-33 | 143.01 | 7.9804 | 0.0053 | up |
| Q8NC42 | RN149 | E3 ubiquitin-protein ligase RNF149 | ILPCKHIFHR | 10 | 1 | 5 | 288 | 6.89E-05 | 90.629 | 7.6233 | 0.0015 | up |
| Q8N1F7 | NUP93 | Nuclear pore complex protein Nup93 | LSPATENKLR | 10 | 1 | 8 | 370 | 8.94E-05 | 93.649 | 7.607 | 0.0411 | up |
| P53990 | IST1 | IST1 homolog | NYNVPYEPDSVVMAEAPPGVETDLIDVGFTDDVKKGGPGR | 40 | 1;2 | 34 | 205 | 9.76E-156 | 122.06 | 7.3512 | 0.0344 | up |
| P53990 | IST1 | IST1 homolog | NYNVPYEPDSVVMAEAPPGVETDLIDVGFTDDVKKGGPGR | 40 | 1;2 | 35 | 206 | 9.76E-156 | 122.06 | 7.3512 | 0.0344 | up |
| Q14247 | SRC8 | Src substrate cortactin | GFGGKFGVQMDR | 12 | 1 | 5 | 124 | 5.14E-05 | 71.03 | 7.0821 | 0.02 | up |
| Q14161 | GIT2 | ARF GTPase-activating protein GIT2 | DLSKQLHSSVR | 11 | 1 | 4 | 135 | 0.0006176 | 61.353 | 6.8755 | 0.0133 | up |
| P48735 | IDHP | Isocitrate dehydrogenase [NADP], mitochondrial | LVPGWTKPITIGR | 13 | 1 | 7 | 166 | 0.0013992 | 45.915 | 6.7625 | 0.0118 | up |
| P22314 | UBA1 | Ubiquitin-like modifier-activating enzyme 1 | QLLHNFPPDQLTSSGAPFWSGPKR | 24 | 1 | 23 | 746 | 1.19E-71 | 120.71 | 6.3578 | 0.0292 | up |
| Q9H6A9 | PCX3 | Pecanex-like protein 3 | GSIQNAKQALR | 11 | 1 | 7 | 1762 | 3.14E-05 | 102.06 | 5.9092 | 0.0023 | up |
| O14493 | CLD4 | Claudin-4 | TDKPYSAKYSAAR | 13 | 2 | 3 | 191 | 5.76E-21 | 124.12 | 5.5859 | 0.0425 | up |
| Q9BSJ8 | ESYT1 | Extended synaptotagmin-1 | IIGVKVHPGQR | 11 | 1 | 5 | 197 | 5.84E-05 | 84.17 | 5.5166 | 0.0054 | up |
| O00232 | PSD12 | 26S proteasome non-ATPase regulatory subunit 12 | LPECAKLAK | 9 | 1 | 6 | 33 | 0.0037754 | 57.859 | 5.3009 | 0.0409 | up |
| Q8WUA4 | TF3C2 | General transcription factor 3C polypeptide 2 | RPYEPINSIKR | 11 | 1 | 10 | 654 | 9.68E-07 | 107.83 | 5.2299 | 0.0351 | up |
| Q92844 | TANK | TRAF family member-associated NF-kappa-B activator | QEALFKPQAKDDINR | 15 | 1;2 | 6 | 195 | 2.94E-05 | 102.06 | 5.1374 | 0.0128 | up |
| P26373 | RL13 | 60S ribosomal protein L13 | NKSTESLQANVQR | 13 | 1 | 2 | 105 | 8.48E-75 | 154.56 | 5.0818 | 0.0236 | up |
| Q9Y5S8 | NOX1 | NADPH oxidase 1 | KQLDHNLTFHK | 11 | 1 | 1 | 92 | 1.08E-53 | 149.94 | 5.0728 | 0.0054 | up |
| P63104 | 1433Z | 14-3-3 protein zeta/delta | MDKNELVQKAK | 11 | 1 | 9 | 9 | 0 | 220.51 | 4.9271 | 0.0132 | up |
| P35579 | MYH9 | Myosin-9 | IGQSKVFFR | 9 | 1 | 5 | 760 | 0.0017927 | 74.929 | 4.7571 | 0.0325 | up |
| P63241 | IF5A1 | Eukaryotic translation initiation factor 5A-1 | GRPCKIVEMSTSK | 13 | 1 | 5 | 39 | 0.0010195 | 66.498 | 4.6673 | 0.0022 | up |
| Q93074 | MED12 | Mediator of RNA polymerase II transcription subunit 12 | LPMQKLPTR | 9 | 1 | 5 | 1867 | 0.0089516 | 55.717 | 4.6525 | 0.0285 | up |
| P15941 | MUC1 | Mucin-1 | KNYGQLDIFPAR | 12 | 1 | 1 | 1189 | 0 | 258 | 4.6158 | 0.0249 | up |
| O43491 | E41L2 | Band 4.1-like protein 2 | IPGEKSVHEGALK | 13 | 1 | 5 | 827 | 4.08E-05 | 71.176 | 4.4886 | 0.0169 | up |
| P48444 | COPD | Coatomer subunit delta | KGVQLQTHPNVDK | 13 | 1 | 1 | 323 | 4.02E-50 | 142.1 | 4.4491 | 0.0102 | up |
| Q14118 | DAG1 | Dystroglycan | KGKLTLEDQATFIK | 14 | 1 | 1 | 780 | 3.20E-05 | 76.073 | 4.4422 | 0.0015 | up |
| Q6UVK1 | CSPG4 | Chondroitin sulfate proteoglycan 4 | TGKHDVQVLTAKPR | 14 | 1;2 | 3 | 2254 | 9.92E-06 | 84.479 | 4.2292 | 0.0481 | up |
| Q63HN8 | RN213 | E3 ubiquitin-protein ligase RNF213 | VTCRPPKEVIDMELSALR | 18 | 1 | 7 | 2136 | 2.37E-81 | 148.7 | 4.1521 | 0.008 | up |
| P56470 | LEG4 | Galectin-4 | KITHNPFGPGQFFDLSIR | 18 | 1 | 1 | 262 | 1.80E-283 | 196.18 | 4.0186 | 0.0231 | up |
| Q15233 | NONO | Non-POU domain-containing octamer-binding protein | PVTVEPMDQLDDEEGLPEKLVIK | 23 | 1 | 19 | 239 | 1.71E-264 | 195.78 | 3.9908 | 0.0042 | up |
| O95786 | DDX58 | Probable ATP-dependent RNA helicase DDX58 | ALVDALKNWIEGNPK | 15 | 1 | 7 | 644 | 9.94E-68 | 140.75 | 3.918 | 0.0327 | up |
| P31948 | STIP1 | Stress-induced-phosphoprotein 1 | NPVIAQKIQK | 10 | 1 | 7 | 530 | 7.66E-19 | 130.22 | 3.8397 | 0.0314 | up |
| P0CG48 | UBC | Polyubiquitin-C | IQDKEGIPPDQQR | 13 | 1 | 4 | 33 | 2.39E-31 | 132.01 | 3.8326 | 0.0151 | up |
| Q8TC12 | RDH11 | Retinol dehydrogenase 11 | GELVAKEIQTTTGNQQVLVR | 20 | 1 | 6 | 83 | 4.96E-25 | 93.765 | 3.8274 | 0.0345 | up |
| O60716 | CTND1 | Catenin delta-1 | KEVHLGACGALK | 12 | 1 | 1 | 422 | 0.0001195 | 83.948 | 3.788 | 0.0208 | up |
| P05362 | ICAM1 | Intercellular adhesion molecule 1 | LQQAQKGTPMKPNTQATPP | 19 | 1 | 6 | 519 | 1.72E-06 | 65.943 | 3.7865 | 0.0244 | up |
| Q9UDY2 | ZO2 | Tight junction protein ZO-2 | HQYSDYDYHSSSEKLK | 16 | 1 | 14 | 434 | 5.75E-41 | 120.7 | 3.6714 | 0.0444 | up |
| Q86YS7 | C2CD5 | C2 domain-containing protein 5 | NQALQTTKTPVEK | 13 | 1 | 8 | 806 | 6.96E-06 | 80.245 | 3.6643 | 0.0376 | up |
| P61421 | VA0D1 | V-type proton ATPase subunit d 1 | GLKAGVLSQADYLNLVQCETLEDLK | 25 | 1 | 3 | 24 | 3.30E-45 | 94.837 | 3.5119 | 0.0013 | up |
| P78504 | JAG1 | Protein jagged-1 | NPIEKHGANTVPIK | 14 | 1 | 5 | 1128 | 2.09E-08 | 85.554 | 3.4234 | 0.0095 | up |
| Q7Z2W4 | ZCCHV | Zinc finger CCCH-type antiviral protein 1 | ARPPSGSSKATDLGGTSQAGTSQR | 24 | 1 | 9 | 314 | 1.32E-45 | 99.059 | 3.4231 | 0.0162 | up |
| P0DP25 | CALM3 | Calmodulin-3 | ADQLTEEQIAEFKEAFSLFDK | 21 | 1 | 13 | 14 | 8.29E-52 | 118.24 | 3.3882 | 0.0197 | up |
| Q15637 | SF01 | Splicing factor 1 | FQRPGDPQSAQDKAR | 15 | 1 | 13 | 306 | 1.61E-43 | 129.37 | 3.3816 | 0.0091 | up |
| P63267 | ACTH | Actin, gamma-enteric smooth muscle | KDLYANNVLSGGTTMYPGIADR | 22 | 1 | 1 | 293 | 2.10E-155 | 160.55 | 3.3517 | 0.0066 | up |
| P13798 | ACPH | Acylamino-acid-releasing enzyme | LKSFNLSALEK | 11 | 1 | 2 | 131 | 0.000476 | 89.355 | 3.3376 | 0.0442 | up |
| Q8TBC4 | UBA3 | NEDD8-activating enzyme E1 catalytic subunit | VPNCNVVPHFNKIQDFNDTFYR | 22 | 1 | 12 | 147 | 0 | 240.17 | 3.2935 | 0.0495 | up |
| Q13347 | EIF3I | Eukaryotic translation initiation factor 3 subunit I | SGEVLVNVKEHSR | 13 | 1 | 9 | 185 | 4.01E-40 | 135.66 | 3.2559 | 0.0206 | up |
| O75367 | H2AY | Core histone macro-H2A.1 | TVKNCLALADDK | 12 | 1 | 3 | 295 | 2.40E-16 | 124.23 | 3.2087 | 0.022 | up |
| P09327 | VILI | Villin-1 | GGVASGMKHVETNSYDVQR | 19 | 1 | 8 | 127 | 4.98E-84 | 136.48 | 3.2001 | 0.0342 | up |
| Q13308 | PTK7 | Inactive tyrosine-protein kinase 7 | DRPSFSEIASALGDSTVDSKP | 21 | 1 | 20 | 1069 | 4.62E-05 | 58.158 | 3.1856 | 0.0135 | up |
| O43707 | ACTN4 | Alpha-actinin-4 | QKASIHEAWTDGK | 13 | 1 | 2 | 421 | 0 | 218.94 | 3.1681 | 0.0062 | up |
| P29353 | SHC1 | SHC-transforming protein 1 | DLFDMKPFEDALR | 13 | 1 | 6 | 462 | 0.0013992 | 45.915 | 3.1464 | 0.0077 | up |
| P36578 | RL4 | 60S ribosomal protein L4 | AAAAAAALQAKSDEK | 15 | 1 | 11 | 364 | 2.96E-94 | 154.56 | 3.1299 | 0.0137 | up |
| O60488 | ACSL4 | Long-chain-fatty-acid--CoA ligase 4 | SDQSYVISFVVPNQKR | 16 | 1 | 15 | 621 | 1.58E-11 | 87.298 | 3.1121 | 0.0174 | up |
| P11142 | HSP7C | Heat shock cognate 71 kDa protein | IQKLLQDFFNGK | 12 | 1 | 3 | 348 | 5.72E-13 | 118.26 | 3.1058 | 0.0167 | up |
| P30086 | PEBP1 | Phosphatidylethanolamine-binding protein 1 | GNDISSGTVLSDYVGSGPPKGTGLHR | 26 | 1 | 20 | 113 | 4.93E-56 | 92.29 | 3.0098 | 0.0077 | up |
| Q9BXS4 | TMM59 | Transmembrane protein 59 | LSIYGDLEFMNEQKLNR | 17 | 1 | 14 | 287 | 1.64E-122 | 156.81 | 2.941 | 0.0494 | up |
| P29372 | 3MG | DNA-3-methyladenine glycosylase | LCQALAINKSFDQR | 14 | 1 | 9 | 229 | 2.10E-08 | 84.365 | 2.932 | 0.0323 | up |
| Q99536 | VAT1 | Synaptic vesicle membrane protein VAT-1 homolog | VLLVPGPEKEN | 11 | 1 | 9 | 391 | 0.0039074 | 65.038 | 2.9004 | 0.0295 | up |
| O95810 | CAVN2 | Caveolae-associated protein 2 | LGTKIVSVER | 10 | 1 | 4 | 261 | 0.0001148 | 86.011 | 2.8922 | 0.041 | up |
| P08034 | CXB1 | Gap junction beta-1 protein | QNEINKLLSEQDGSLKDILR | 20 | 1;2 | 6 | 250 | 1.67E-19 | 109.42 | 2.8387 | 0.0394 | up |
| O43760 | SNG2 | Synaptogyrin-2 | MESGAYGAAKAGGSFDLR | 18 | 1 | 10 | 10 | 3.57E-204 | 179.67 | 2.8272 | 0.0233 | up |
| P78527 | PRKDC | DNA-dependent protein kinase catalytic subunit | NNITQKLLQDFNR | 13 | 1 | 6 | 2835 | 1.09E-60 | 145.17 | 2.7511 | 0.0401 | up |
| P40305 | IFI27 | Interferon alpha-inducible protein 27, mitochondrial | MEASALTSSAVTSVAKVVR | 19 | 1 | 16 | 16 | 1.23E-219 | 180.88 | 2.7419 | 0.0039 | up |
| Q13873 | BMPR2 | Bone morphogenetic protein receptor type-2 | SNLKQVETGVAK | 12 | 1 | 4 | 782 | 0.0002432 | 93.598 | 2.7024 | 0.0436 | up |
| Q14517 | FAT1 | Protocadherin Fat 1 | DKHLGPATAFLQR | 13 | 1 | 2 | 4219 | 2.60E-15 | 103.88 | 2.6532 | 0.0354 | up |
| Q8IY95 | TM192 | Transmembrane protein 192 | AKPEPDILEEEKIYAYPSNITSETGFR | 27 | 1;2 | 2 | 201 | 2.39E-47 | 81.949 | 2.6284 | 0.0126 | up |
| P08758 | ANXA5 | Annexin A5 | AMKGLGTDEESILTLLTSR | 19 | 1 | 3 | 29 | 5.09E-25 | 97.579 | 2.6079 | 0.0183 | up |
| P35749 | MYH11 | Myosin-11 | SGKLDAFLVLEQLR | 14 | 1 | 3 | 689 | 4.26E-39 | 129.7 | 2.5918 | 0.0318 | up |
| P60174 | TPIS | Triosephosphate isomerase | TATPQQAQEVHEKLR | 15 | 1 | 13 | 225 | 2.20E-37 | 136.27 | 2.5125 | 0.0123 | up |
| O14686 | KMT2D | Histone-lysine N-methyltransferase 2D | SEAGHLLLQKLLR | 13 | 1 | 10 | 4465 | 2.07E-06 | 88.441 | 2.4733 | 0.0378 | up |
| O75674 | TM1L1 | TOM1-like protein 1 | EVYLDLVKK | 9 | 1 | 9 | 149 | 0.0040504 | 83.614 | 2.4534 | 0.0372 | up |
| P62330 | ARF6 | ADP-ribosylation factor 6 | FNVWDVGGQDKIRPLWR | 17 | 1 | 11 | 69 | 4.45E-22 | 111.63 | 2.4372 | 0.0287 | up |
| P50443 | S26A2 | Sulfate transporter | SALYKQTVNPILIK | 14 | 1 | 5 | 601 | 4.75E-11 | 107.15 | 2.4145 | 0.0374 | up |
| P17302 | CXA1 | Gap junction alpha-1 protein | GKSDPYHATSGALSPAK | 17 | 1 | 2 | 243 | 1.08E-69 | 137.17 | 2.3998 | 0.0155 | up |
| O14638 | ENPP3 | Ectonucleotide pyrophosphatase/phosphodiesterase family member 3 | MESTLTLATEQPVKK | 15 | 1 | 14 | 14 | 5.89E-81 | 147.2 | 2.3905 | 0.0058 | up |
| P61088 | UBE2N | Ubiquitin-conjugating enzyme E2 N | ICLDILKDK | 9 | 1 | 7 | 92 | 6.75E-35 | 154.68 | 2.3904 | 0.0141 | up |
| Q8NI35 | INADL | InaD-like protein | DCKGLGFSILDYQDPLDPTR | 20 | 1 | 3 | 694 | 9.15E-27 | 101.15 | 2.3836 | 0.0046 | up |
| Q15907 | RB11B | Ras-related protein Rab-11B | AFAEKNNLSFIETSALDSTNVEEAFK | 26 | 1 | 5 | 145 | 0 | 223.51 | 2.3542 | 0.0139 | up |
| P18085 | ARF4 | ADP-ribosylation factor 4 | QDLPNAMAISEMTDKLGLQSLR | 22 | 1 | 15 | 142 | 2.73E-51 | 114.4 | 2.3414 | 0.0147 | up |
| P55072 | TERA | Transitional endoplasmic reticulum ATPase | ASGADSKGDDLSTAILK | 17 | 1;2 | 7 | 8 | 0 | 229.16 | 2.3273 | 0.0073 | up |
| Q96BH1 | RNF25 | E3 ubiquitin-protein ligase RNF25 | AGLGTAMLYELIEKGK | 16 | 1 | 14 | 120 | 2.34E-11 | 96.847 | 2.2909 | 0.0045 | up |
| O95810 | CAVN2 | Caveolae-associated protein 2 | VLIFQEENEIPASVFVKQPVSGAVEGK | 27 | 1 | 17 | 173 | 1.62E-59 | 100.94 | 2.242 | 0.0269 | up |
| Q86VP1 | TAXB1 | Tax1-binding protein 1 | ESVITHFKEEIGR | 13 | 1 | 8 | 319 | 0.0002736 | 63.537 | 2.2381 | 0.0119 | up |
| P31948 | STIP1 | Stress-induced-phosphoprotein 1 | TVDLKPDWGK | 10 | 1 | 5 | 68 | 0.0164307 | 53.17 | 2.0751 | 0.0251 | up |
| P16144 | ITB4 | Integrin beta-4 | KQDHTIVDTVLMAPR | 15 | 1 | 1 | 878 | 4.25E-10 | 80.96 | 2.0718 | 0.0265 | up |
| P21860 | ERBB3 | Receptor tyrosine-protein kinase erbB-3 | LAEVPDLLEKGER | 13 | 1 | 10 | 926 | 1.16E-05 | 97.431 | 2.0502 | 0.0241 | up |
| Q6ZNB6 | NFXL1 | NF-X1-type zinc finger protein NFXL1 | EKGSVGAVPSGTSPGGVATTAAAGSR | 26 | 1 | 2 | 39 | 1.83E-89 | 107.74 | 0.0112 | 0.0007 | down |
| P52272 | HNRPM | Heterogeneous nuclear ribonucleoprotein M | INEILSNALKR | 11 | 1 | 10 | 381 | 1.29E-182 | 185.84 | 0.0139 | 0.0004 | down |
| P26038 | MOES | Moesin | YGDFNKEVHK | 10 | 1 | 6 | 139 | 7.21E-07 | 109.86 | 0.0439 | 0.0342 | down |
| P05534 | 1A24 | HLA class I histocompatibility antigen, A-24 alpha chain | KGGSYSQAASSDSAQGSDVSLTACKV | 26 | 1;2 | 1 | 340 | 0 | 234.66 | 0.0493 | 0.0064 | down |
| P05534 | 1A24 | HLA class I histocompatibility antigen, A-24 alpha chain | KGGSYSQAASSDSAQGSDVSLTACKV | 26 | 1;2 | 25 | 364 | 1.00E-138 | 143.95 | 0.0543 | 0.0128 | down |
| P46019 | KPB2 | Phosphorylase b kinase regulatory subunit alpha, liver isoform | SLNLVDSPQPLLEKVPESDFQWPR | 24 | 1;2 | 14 | 742 | 1.49E-44 | 92.492 | 0.0579 | 0.0085 | down |
| Q5T7N2 | LITD1 | LINE-1 type transposase domain-containing protein 1 | AVLGGKATIPEVK | 13 | 1 | 6 | 84 | 0.0080532 | 46.158 | 0.0601 | 0.0331 | down |
| Q92973 | TNPO1 | Transportin-1 | KCSAAALDVLANVYR | 15 | 1 | 1 | 385 | 0 | 210.87 | 0.0791 | 0.0012 | down |
| Q14152 | EIF3A | Eukaryotic translation initiation factor 3 subunit A | LLDMDGIIVEKQR | 13 | 1 | 11 | 351 | 4.96E-25 | 126.41 | 0.1054 | 0.0092 | down |
| O75367 | H2AY | Core histone macro-H2A.1 | GVTIASGGVLPNIHPELLAKKR | 22 | 1 | 20 | 116 | 0 | 256.96 | 0.1142 | 0.011 | down |
| Q8NG31 | KNL1 | Kinetochore scaffold 1 | NIKDVQSPGFLNEPLSSK | 18 | 1 | 3 | 1072 | 6.16E-160 | 166.9 | 0.1354 | 0.0065 | down |
| Q86WI3 | NLRC5 | Protein NLRC5 | KCGDAFAEALSR | 12 | 1 | 1 | 697 | 0.0021588 | 45.077 | 0.1364 | 0.0205 | down |
| P55036 | PSMD4 | 26S proteasome non-ATPase regulatory subunit 4 | AAAASAAEAGIATTGTEDSDDALLKMTISQQEFGR | 35 | 1 | 25 | 262 | 2.06E-86 | 97.063 | 0.1365 | 0.0059 | down |
| O75110 | ATP9A | Probable phospholipid-transporting ATPase IIA | DSKIPGTVVR | 10 | 1 | 3 | 367 | 0.011821 | 56.916 | 0.1414 | 0.0047 | down |
| P30305 | MPIP2 | M-phase inducer phosphatase 2 | ELIGDYSKAFLLQTVDGK | 18 | 1 | 8 | 398 | 2.84E-11 | 75.961 | 0.153 | 0.0008 | down |
| P06493 | CDK1 | Cyclin-dependent kinase 1 | KPLFHGDSEIDQLFR | 15 | 1 | 1 | 201 | 0 | 227.53 | 0.1566 | 0.0066 | down |
| P15924 | DESP | Desmoplakin | DYELQLASYTSGLETLLNIPIKR | 23 | 1 | 22 | 981 | 9.75E-141 | 149.47 | 0.1604 | 0.0132 | down |
| Q9BRK3 | MXRA8 | Matrix remodeling-associated protein 8 | SEDIQLDYKNNILKER | 16 | 1;2 | 9 | 410 | 6.60E-132 | 160.59 | 0.1671 | 0.0356 | down |
| Q9UKK3 | PARP4 | Poly [ADP-ribose] polymerase 4 | EVLQNHHSKSPVDVLQIFR | 19 | 1 | 9 | 406 | 0.0001153 | 54.281 | 0.1762 | 0.0059 | down |
| P51587 | BRCA2 | Breast cancer type 2 susceptibility protein | EGNTQIKEDLSDLTFLEVAK | 20 | 1 | 7 | 1381 | 1.51E-05 | 57.338 | 0.1971 | 0.0049 | down |
| Q15154 | PCM1 | Pericentriolar material 1 protein | WVSELSYVEEKEQWQEQINQLK | 22 | 1 | 11 | 996 | 1.11E-51 | 117.26 | 0.2009 | 0.0467 | down |
| Q76MJ5 | ERN2 | Serine/threonine-protein kinase/endoribonuclease IRE2 | LQSPSKQAQPLDDPEAEQLTVVGK | 24 | 1 | 6 | 499 | 2.17E-50 | 106.49 | 0.2009 | 0.0037 | down |
| Q08117 | AES | Amino-terminal enhancer of split | AKQVTAPELNSIIR | 14 | 1 | 2 | 112 | 2.31E-15 | 113.22 | 0.2019 | 0.0013 | down |
| Q9NS91 | RAD18 | E3 ubiquitin-protein ligase RAD18 | VDCPVCGVNIPESHINKHLDSCLSR | 25 | 1 | 17 | 218 | 1.16E-125 | 143.15 | 0.2029 | 0.0015 | down |
| P06737 | PYGL | Glycogen phosphorylase, liver form | VLYPNDNFFEGKELR | 15 | 1 | 12 | 290 | 5.90E-20 | 110.38 | 0.2076 | 0.0026 | down |
| Q8WVC6 | DCAKD | Dephospho-CoA kinase domain-containing protein | KVLGDLIFNQPDR | 13 | 1 | 1 | 67 | 4.23E-17 | 114.84 | 0.211 | 0.0054 | down |
| P84077 | ARF1 | ADP-ribosylation factor 1 | QDLPNAMNAAEITDKLGLHSLR | 22 | 1 | 15 | 142 | 3.46E-171 | 162.15 | 0.2201 | 0.0435 | down |
| Q9Y4L5 | RN115 | E3 ubiquitin-protein ligase RNF115 | GEVSPKLPEYICPR | 14 | 1 | 6 | 32 | 8.26E-06 | 74.46 | 0.2222 | 0.0063 | down |
| Q99973 | TEP1 | Telomerase protein component 1 | LLPKSAESLHPGQTQVLIIDGADR | 24 | 1 | 4 | 1250 | 2.35E-05 | 44.729 | 0.2237 | 0.002 | down |
| P11142 | HSP7C | Heat shock cognate 71 kDa protein | ELNKSINPDEAVAYGAAVQAAILSGDK | 27 | 1 | 4 | 361 | 8.14E-56 | 89.365 | 0.2247 | 0.0015 | down |
| Q14139 | UBE4A | Ubiquitin conjugation factor E4 A | DLADYASKNLEAMNPPLFLR | 20 | 1 | 8 | 774 | 0.0001291 | 51.606 | 0.2251 | 0.0151 | down |
| Q96J92 | WNK4 | Serine/threonine-protein kinase WNK4 | IGDLGLATLKR | 11 | 1 | 10 | 375 | 1.21E-10 | 107.66 | 0.2285 | 0.0146 | down |
| P49327 | FAS | Fatty acid synthase | VVVQVLAEEPEAVLKGAK | 18 | 1 | 18 | 1869 | 3.04E-19 | 103.34 | 0.2294 | 0.0398 | down |
| O60884 | DNJA2 | DnaJ homolog subfamily A member 2 | EYHPDKNPNAGDKFK | 15 | 1 | 15 | 48 | 5.95E-09 | 79.633 | 0.2325 | 0.0078 | down |
| Q6ZRV2 | FA83H | Protein FAM83H | PQGLFEKLR | 9 | 1 | 7 | 475 | 1.68E-55 | 88.935 | 0.2349 | 0.0035 | down |
| Q9BTW9 | TBCD | Tubulin-specific chaperone D | GELEKLFPR | 9 | 1 | 5 | 947 | 0.001817 | 107.43 | 0.2368 | 0.0053 | down |
| Q96RT7 | GCP6 | Gamma-tubulin complex component 6 | DYFLNNKHVGR | 11 | 1 | 7 | 137 | 0.0005175 | 62.303 | 0.238 | 0.0404 | down |
| Q9UKD2 | MRT4 | mRNA turnover protein 4 homolog | QLGLPTALKR | 10 | 1 | 9 | 163 | 0.0268531 | 67.032 | 0.2396 | 0.0266 | down |
| Q96JI7 | SPTCS | Spatacsin | SVLDSFLKYDCK | 12 | 1 | 8 | 835 | 0.0084613 | 48.283 | 0.2432 | 0.0126 | down |
| Q7Z333 | SETX | Probable helicase senataxin | VIELGLLESPDIYTSSVLEKGK | 22 | 1 | 22 | 220 | 2.01E-171 | 162.4 | 0.2435 | 0.0035 | down |
| Q8IXQ6 | PARP9 | Poly [ADP-ribose] polymerase 9 | SILQQAGVEMKSEFLATK | 18 | 1 | 11 | 361 | 1.11E-22 | 107.98 | 0.2447 | 0.0327 | down |
| Q01968 | OCRL | Inositol polyphosphate 5-phosphatase OCRL-1 | AFVDFNEGEIKFIPTYK | 17 | 1 | 11 | 472 | 0.000387 | 44.807 | 0.248 | 0.0156 | down |
| Q8NB46 | ANR52 | Serine/threonine-protein phosphatase 6 regulatory ankyrin repeat subunit C | SLLSQKENINVLDQER | 16 | 1 | 6 | 31 | 3.78E-50 | 130.07 | 0.2481 | 0.0037 | down |
| O00264 | PGRC1 | Membrane-associated progesterone receptor component 1 | KFYGPEGPYGVFAGR | 15 | 1 | 1 | 105 | 2.63E-13 | 102.9 | 0.2489 | 0.0003 | down |
| Q8IUF1 | CBWD2 | COBW domain-containing protein 2 | SQQVIVQGVHELYDLEETPVSWKDDTER | 28 | 1 | 23 | 348 | 1.03E-15 | 63.573 | 0.2519 | 0.0082 | down |
| Q9H000 | MKRN2 | Probable E3 ubiquitin-protein ligase makorin-2 | YFEQGKGTCPFGSK | 14 | 1 | 6 | 334 | 4.37E-07 | 80.688 | 0.2577 | 0.0065 | down |
| Q92922 | SMRC1 | SWI/SNF complex subunit SMARCC1 | NKEKPVDLQNFGLR | 14 | 1 | 2 | 590 | 4.87E-201 | 181.66 | 0.259 | 0.0249 | down |
| Q86YV9 | HPS6 | Hermansky-Pudlak syndrome 6 protein | ALQLQLDGNGKLR | 13 | 1 | 11 | 534 | 6.41E-16 | 99.802 | 0.2643 | 0.012 | down |
| P33991 | MCM4 | DNA replication licensing factor MCM4 | LGEINVIGEPFLNVNCEHIKSFDK | 24 | 1 | 20 | 216 | 1.03E-45 | 98.269 | 0.2684 | 0.0214 | down |
| P51787 | KCNQ1 | Potassium voltage-gated channel subfamily KQT member 1 | LDQSIGKPSLFISVSEKSK | 19 | 1 | 19 | 581 | 1.28E-93 | 140.22 | 0.269 | 0.0481 | down |
| O75151 | PHF2 | Lysine-specific demethylase PHF2 | KQALAEHEDELPEHFKPSQLIK | 22 | 1 | 1 | 412 | 3.69E-47 | 93.226 | 0.2709 | 0.0182 | down |
| Q6P996 | PDXD1 | Pyridoxal-dependent decarboxylase domain-containing protein 1 | KGIQEAQVELQK | 12 | 1 | 1 | 615 | 5.08E-12 | 110.84 | 0.2729 | 0.0284 | down |
| O95785 | WIZ | Protein Wiz | FVGNIYTLKCR | 11 | 1 | 9 | 1597 | 7.28E-10 | 116.25 | 0.2789 | 0.0222 | down |
| P01024 | CO3 | Complement C3 | KVFLDCCNYITELR | 14 | 1 | 1 | 722 | 2.31E-32 | 125.48 | 0.2796 | 0.0114 | down |
| Q9NSD9 | SYFB | Phenylalanine--tRNA ligase beta subunit | AAGASDVVLYKIDVPANR | 18 | 1 | 11 | 64 | 1.13E-13 | 78.285 | 0.2834 | 0.0325 | down |
| P39019 | RS19 | 40S ribosomal protein S19 | VLQALEGLKMVEK | 13 | 1 | 9 | 111 | 1.48E-15 | 87.001 | 0.2858 | 0.0149 | down |
| P78371 | TCPB | T-complex protein 1 subunit beta | ASLSLAPVNIFKAGADEER | 19 | 1 | 12 | 13 | 8.93E-219 | 178.81 | 0.2884 | 0.0003 | down |
| P15531 | NDKA | Nucleoside diphosphate kinase A | TFIAIKPDGVQR | 12 | 1 | 6 | 12 | 8.61E-13 | 125.96 | 0.2905 | 0.0103 | down |
| O43447 | PPIH | Peptidyl-prolyl cis-trans isomerase H | GPFADENFKLR | 11 | 1 | 9 | 101 | 0.0008193 | 73.931 | 0.2922 | 0.0027 | down |
| Q8WUX1 | S38A5 | Sodium-coupled neutral amino acid transporter 5 | GPAPGSKPVQFMDFEGK | 17 | 1 | 7 | 37 | 1.85E-18 | 99.481 | 0.2934 | 0.0345 | down |
| P52272 | HNRPM | Heterogeneous nuclear ribonucleoprotein M | RGEIIAKQGGGGGGGSVPGIER | 22 | 1 | 7 | 388 | 0 | 218.85 | 0.2955 | 0.0464 | down |
| P12004 | PCNA | Proliferating cell nuclear antigen | ILKCAGNEDIITLR | 14 | 1 | 3 | 80 | 2.29E-155 | 174.73 | 0.2997 | 0.0036 | down |
| P62424 | RL7A | 60S ribosomal protein L7a | GALAKLVEAIR | 11 | 1 | 5 | 217 | 5.11E-53 | 147.51 | 0.3046 | 0.0013 | down |
| P62241 | RS8 | 40S ribosomal protein S8 | NAKISSLLEEQFQQGK | 16 | 1 | 3 | 157 | 2.74E-50 | 131.35 | 0.3124 | 0.0045 | down |
| P01911 | 2B1F | HLA class II histocompatibility antigen, DRB1-15 beta chain | NQKGHSGLQPTGFLS | 15 | 1 | 3 | 254 | 8.35E-94 | 152.97 | 0.3144 | 0.0223 | down |
| Q6P996 | PDXD1 | Pyridoxal-dependent decarboxylase domain-containing protein 1 | KPVIYLSAAAR | 11 | 1 | 1 | 173 | 4.80E-27 | 117.07 | 0.3145 | 0.0356 | down |
| Q14149 | MORC3 | MORC family CW-type zinc finger protein 3 | STNQQTATDVSTSSNIEESVNHMDGESLKLR | 31 | 1 | 29 | 891 | 1.84E-112 | 128.17 | 0.3199 | 0.0003 | down |
| P35249 | RFC4 | Replication factor C subunit 4 | KSLEGADLPNLLFYGPPGTGK | 21 | 1 | 1 | 64 | 8.48E-13 | 69.148 | 0.3223 | 0.0127 | down |
| O75113 | N4BP1 | NEDD4-binding protein 1 | LEEFLQKEVCLR | 12 | 1 | 7 | 773 | 2.59E-05 | 80.596 | 0.3231 | 0.0289 | down |
| Q9BRK3 | MXRA8 | Matrix remodeling-associated protein 8 | GKDVNLAEFAVAAGDQMLYR | 20 | 1 | 2 | 383 | 2.28E-95 | 143.28 | 0.3244 | 0.0071 | down |
| P78371 | TCPB | T-complex protein 1 subunit beta | DASLMVTNDGATILKNIGVDNPAAK | 25 | 1 | 15 | 72 | 5.47E-141 | 148.26 | 0.3256 | 0.0336 | down |
| Q9UHP3 | UBP25 | Ubiquitin carboxyl-terminal hydrolase 25 | AVEILKDAFK | 10 | 1 | 6 | 250 | 0.0001889 | 100.45 | 0.3285 | 0.0234 | down |
| Q6ZNJ1 | NBEL2 | Neurobeachin-like protein 2 | LIQNSKLYLQSR | 12 | 1 | 6 | 344 | 1.54E-11 | 112.84 | 0.3353 | 0.0091 | down |
| P20700 | LMNB1 | Lamin-B1 | AKLQIELGK | 9 | 1 | 2 | 102 | 0.0006884 | 86.794 | 0.3356 | 0.0342 | down |
| P52788 | SPSY | Spermine synthase | LVEYDIDEVVYDEDSPYQNIKILHSK | 26 | 1 | 21 | 151 | 7.33E-76 | 114.72 | 0.3403 | 0.0269 | down |
| Q03188 | CENPC | Centromere protein C | TLDTPFFSTGKLILGPQEEK | 20 | 1 | 11 | 871 | 1.32E-23 | 83.386 | 0.3413 | 0.0067 | down |
| P28066 | PSA5 | Proteasome subunit alpha type-5 | SMTLKEAIK | 9 | 1 | 5 | 192 | 2.45E-08 | 70.912 | 0.3414 | 0.0437 | down |
| Q14258 | TRI25 | E3 ubiquitin/ISG15 ligase TRIM25 | LPTFGAPEQLVDLKQAGLEAAAK | 23 | 1 | 14 | 416 | 0 | 196.71 | 0.343 | 0.0029 | down |
| O00487 | PSDE | 26S proteasome non-ATPase regulatory subunit 14 | AVAVVVDPIQSVKGK | 15 | 1 | 15 | 154 | 2.38E-12 | 95.018 | 0.3447 | 0.0376 | down |
| P31689 | DNJA1 | DnaJ homolog subfamily A member 1 | QISQAYEVLSDAKK | 14 | 1;2 | 14 | 60 | 2.31E-32 | 125.48 | 0.3447 | 0.0304 | down |
| Q13535 | ATR | Serine/threonine-protein kinase ATR | TLQVLLPDLAAKASPAASALIR | 22 | 1 | 12 | 1005 | 3.80E-13 | 69.737 | 0.3454 | 0.0017 | down |
| O75083 | WDR1 | WD repeat-containing protein 1 | YAPSGFYIASGDVSGKLR | 18 | 1 | 16 | 81 | 2.36E-10 | 71.98 | 0.3456 | 0.0105 | down |
| P42685 | FRK | Tyrosine-protein kinase FRK | IQVPAPFDLSYKTVDQWEIDR | 21 | 1 | 12 | 222 | 1.84E-06 | 61.102 | 0.347 | 0.0022 | down |
| Q68DQ2 | CRBG3 | Very large A-kinase anchor protein | NLLVDPNSMNVSCLLEDKAR | 20 | 1 | 18 | 1304 | 0.0003058 | 47.754 | 0.3479 | 0.0075 | down |
| Q86VI3 | IQGA3 | Ras GTPase-activating-like protein IQGAP3 | VLAVSLINEALDKGSPEK | 18 | 1 | 13 | 537 | 8.03E-08 | 65.172 | 0.3489 | 0.0231 | down |
| P78545 | ELF3 | ETS-related transcription factor Elf-3 | NKYDASAIDFSR | 12 | 1 | 2 | 84 | 8.19E-12 | 108.98 | 0.3506 | 0.0162 | down |
| Q99829 | CPNE1 | Copine-1 | NCSSPEFSKTLQLEYR | 16 | 1 | 9 | 60 | 5.24E-13 | 99.941 | 0.3512 | 0.0046 | down |
| P06493 | CDK1 | Cyclin-dependent kinase 1 | DLKPQNLLIDDK | 12 | 1;2 | 3 | 130 | 9.14E-05 | 80.239 | 0.3528 | 0.01 | down |
| P23921 | RIR1 | Ribonucleoside-diphosphate reductase large subunit | DFSYNYFGFKTLER | 14 | 1 | 10 | 149 | 4.84E-08 | 89.301 | 0.3543 | 0.012 | down |
| Q5TDH0 | DDI2 | Protein DDI1 homolog 2 | NPPLAEALLSGDLEKFSR | 18 | 1 | 15 | 170 | 1.46E-228 | 184.87 | 0.3547 | 0.003 | down |
| P55196 | AFAD | Afadin | GAKEIILDDDECPLQIFR | 18 | 1 | 3 | 315 | 7.87E-06 | 60.918 | 0.3577 | 0.0416 | down |
| O60784 | TOM1 | Target of Myb protein 1 | EVKYEAPQATDGLAGALDAR | 20 | 1 | 3 | 385 | 4.72E-25 | 94.01 | 0.358 | 0.0239 | down |
| Q9Y5V3 | MAGD1 | Melanoma-associated antigen D1 | KLLTYEFVK | 9 | 1 | 1 | 605 | 0.0011395 | 103.92 | 0.3616 | 0.0105 | down |
| O96019 | ACL6A | Actin-like protein 6A | LKLIANNTTVER | 12 | 1 | 2 | 379 | 0.0024993 | 57.598 | 0.365 | 0.0216 | down |
| P78536 | ADA17 | Disintegrin and metalloproteinase domain-containing protein 17 | IIKPFPAPQTPGR | 13 | 1 | 3 | 728 | 5.15E-06 | 89.805 | 0.3656 | 0.0142 | down |
| O95994 | AGR2 | Anterior gradient protein 2 homolog | GWGDQLIWTQTYEEALYKSK | 20 | 1 | 20 | 66 | 0.000185 | 50.387 | 0.3708 | 0.0212 | down |
| P20839 | IMDH1 | Inosine-5'-monophosphate dehydrogenase 1 | GKLPIVNDCDELVAIIAR | 18 | 1 | 2 | 208 | 1.56E-160 | 169.92 | 0.3721 | 0.0004 | down |
| P05141 | ADT2 | ADP/ATP translocase 2 | YKQIFLGGVDK | 11 | 1 | 2 | 96 | 0.0008193 | 73.931 | 0.3741 | 0.003 | down |
| Q9UIA9 | XPO7 | Exportin-7 | GSSSYSQLLAATCLTKLVSR | 20 | 1 | 16 | 69 | 1.99E-26 | 98.507 | 0.3742 | 0.0095 | down |
| P20839 | IMDH1 | Inosine-5'-monophosphate dehydrogenase 1 | DSQKQLLCGAAVGTR | 15 | 1 | 4 | 242 | 1.75E-11 | 84.17 | 0.3761 | 0.0093 | down |
| O60499 | STX10 | Syntaxin-10 | PAAQKSPSDLLDASAVSATSR | 21 | 1 | 5 | 131 | 1.08E-18 | 73.809 | 0.3765 | 0.0071 | down |
| P11216 | PYGB | Glycogen phosphorylase, brain form | APNDFKLQDFNVGDYIEAVLDR | 22 | 1 | 6 | 254 | 7.40E-83 | 131.11 | 0.3766 | 0.0487 | down |
| Q9NVJ2 | ARL8B | ADP-ribosylation factor-like protein 8B | QLIEKMNLSAIQDR | 14 | 1 | 5 | 146 | 8.28E-28 | 119.68 | 0.3791 | 0.0053 | down |
| Q9Y5B0 | CTDP1 | RNA polymerase II subunit A C-terminal domain phosphatase | DECIDPFSKTGNLR | 14 | 1 | 9 | 283 | 2.68E-10 | 104.17 | 0.3836 | 0.0476 | down |
| P49591 | SYSC | Serine--tRNA ligase, cytoplasmic | DEWLRPEDLPIKYAGLSTCFR | 21 | 1 | 12 | 293 | 8.21E-25 | 78.021 | 0.3852 | 0.0407 | down |
| Q5MIZ7 | P4R3B | Serine/threonine-protein phosphatase 4 regulatory subunit 3B | YITKGNLFEPVINALLDNGTR | 21 | 1 | 4 | 611 | 2.08E-25 | 80.638 | 0.3861 | 0.0339 | down |
| P50991 | TCPD | T-complex protein 1 subunit delta | MIQDGKGDVTITNDGATILK | 20 | 1 | 6 | 65 | 2.05E-25 | 96.666 | 0.3868 | 0.0408 | down |
| P52272 | HNRPM | Heterogeneous nuclear ribonucleoprotein M | GCGVVKFESPEVAER | 15 | 1 | 6 | 698 | 1.32E-14 | 105.4 | 0.3879 | 0.0378 | down |
| P52597 | HNRPF | Heterogeneous nuclear ribonucleoprotein F | YIEVFKSHR | 9 | 1 | 6 | 87 | 5.72E-07 | 123.96 | 0.3887 | 0.0056 | down |
| P29401 | TKT | Transketolase | SVPTSTVFYPSDGVATEKAVELAANTK | 27 | 1 | 18 | 456 | 8.65E-20 | 68.375 | 0.3889 | 0.0387 | down |
| Q92621 | NU205 | Nuclear pore complex protein Nup205 | KLLDIEGLYSK | 11 | 1 | 1 | 1978 | 1.56E-40 | 79.237 | 0.3916 | 0.0016 | down |
| Q92621 | NU205 | Nuclear pore complex protein Nup205 | SGLAIVSQHDLDQLQADAINAFGESLQKK | 29 | 1 | 28 | 1977 | 1.56E-40 | 79.237 | 0.3916 | 0.0016 | down |
| P62906 | RL10A | 60S ribosomal protein L10a | KYDAFLASESLIK | 13 | 1 | 1 | 106 | 3.96E-20 | 120.12 | 0.3917 | 0.0331 | down |
| Q86XI2 | CNDG2 | Condensin-2 complex subunit G2 | STGILGVCKITSK | 13 | 1 | 9 | 402 | 0.0032298 | 57.598 | 0.3949 | 0.0399 | down |
| P51570 | GALK1 | Galactokinase | SLETSLVPLSDPKLAVLITNSNVR | 24 | 1 | 13 | 217 | 3.93E-11 | 64.723 | 0.3976 | 0.0453 | down |
| O75843 | AP1G2 | AP-1 complex subunit gamma-like 2 | DLAPEVEKLLLQPSPYVR | 18 | 1 | 8 | 145 | 1.93E-21 | 106.35 | 0.3982 | 0.0468 | down |
| P52948 | NUP98 | Nuclear pore complex protein Nup98-Nup96 | ETFLTQKLR | 9 | 1 | 7 | 1608 | 0.0091915 | 68.915 | 0.3983 | 0.0213 | down |
| Q9ULC5 | ACSL5 | Long-chain-fatty-acid--CoA ligase 5 | LIFAKIQDSLGGR | 13 | 1 | 5 | 404 | 5.28E-16 | 100.25 | 0.4 | 0.0431 | down |
| Q8NG31 | KNL1 | Kinetochore scaffold 1 | DNSCVQEIAEKQALAVGNK | 19 | 1 | 11 | 1240 | 1.13E-24 | 90.754 | 0.4002 | 0.0419 | down |
| Q14254 | FLOT2 | Flotillin-2 | MALVLEALPQIAAKIAAPLTK | 21 | 1 | 14 | 370 | 4.61E-44 | 110.22 | 0.4021 | 0.0087 | down |
| P13569 | CFTR | Cystic fibrosis transmembrane conductance regulator | FAEKDNIVLGEGGITLSGGQR | 21 | 1 | 4 | 536 | 4.38E-25 | 78.021 | 0.405 | 0.0432 | down |
| P68104 | EF1A1 | Elongation factor 1-alpha 1 | SGDAAIVDMVPGKPMCVESFSDYPPLGR | 28 | 1;2 | 13 | 408 | 6.08E-140 | 141.78 | 0.4057 | 0.0284 | down |
| Q8TAQ2 | SMRC2 | SWI/SNF complex subunit SMARCC2 | DIGEGNLSTAAAAALAAAAVKAK | 23 | 1 | 23 | 874 | 9.51E-39 | 85.146 | 0.4057 | 0.0012 | down |
| P49458 | SRP09 | Signal recognition particle 9 kDa protein | AAEKLYLADPMK | 12 | 1 | 4 | 16 | 0.0001085 | 92.062 | 0.4094 | 0.0001 | down |
| Q14202 | ZMYM3 | Zinc finger MYM-type protein 3 | IKEDILACSAAELNYGLAQFVR | 22 | 1 | 2 | 1100 | 6.10E-31 | 86.479 | 0.4128 | 0.0034 | down |
| O14562 | UBFD1 | Ubiquitin domain-containing protein UBFD1 | DTVLGKWQYF | 10 | 1 | 6 | 305 | 8.04E-31 | 145.04 | 0.4169 | 0.0014 | down |
| Q10570 | CPSF1 | Cleavage and polyadenylation specificity factor subunit 1 | KIGTTPDIILDDLLETDR | 18 | 1 | 1 | 1421 | 4.69E-18 | 88.73 | 0.4169 | 0.0057 | down |
| P07900 | HS90A | Heat shock protein HSP 90-alpha | LDSGKELHINLIPNK | 15 | 1 | 5 | 74 | 1.12E-34 | 122.42 | 0.4196 | 0.0325 | down |
| P23396 | RS3 | 40S ribosomal protein S3 | GGKPEPPAMPQPVPTA | 16 | 1 | 3 | 230 | 3.06E-11 | 95.573 | 0.4241 | 0.0258 | down |
| P39060 | COIA1 | Collagen alpha-1(XVIII) chain | DELLFPSWEALFSGSEGPLKPGAR | 24 | 1 | 20 | 1666 | 1.71E-55 | 109.66 | 0.4251 | 0.0499 | down |
| P25815 | S100P | Protein S100-P | YSGSEGSTQTLTKGELK | 17 | 1 | 13 | 30 | 2.11E-80 | 143.61 | 0.4258 | 0.0026 | down |
| P24928 | RPB1 | DNA-directed RNA polymerase II subunit RPB1 | INISQVIAVVGQQNVEGKR | 19 | 1 | 18 | 796 | 4.30E-84 | 136.48 | 0.4267 | 0.0128 | down |
| Q8TAQ2 | SMRC2 | SWI/SNF complex subunit SMARCC2 | DIGEGNLSTAAAAALAAAAVKAK | 23 | 1 | 21 | 872 | 9.51E-39 | 85.146 | 0.4294 | 0.0395 | down |
| O75592 | MYCB2 | E3 ubiquitin-protein ligase MYCBP2 | DDKASFYGFK | 10 | 1 | 3 | 2132 | 0.0031488 | 72.547 | 0.4318 | 0.0204 | down |
| P0DN76 | U2AF5 | Splicing factor U2AF 35 kDa subunit-like protein | AEYLASIFGTEKDK | 14 | 1 | 12 | 13 | 4.92E-29 | 122.13 | 0.4339 | 0.0031 | down |
| Q99650 | OSMR | Oncostatin-M-specific receptor subunit beta | SLTETELTKPNYLYLLPTEK | 20 | 1 | 9 | 834 | 4.73E-06 | 60.768 | 0.4343 | 0.0025 | down |
| Q9UI10 | EI2BD | Translation initiation factor eIF-2B subunit delta | FLNKEITSVGSSK | 13 | 1 | 4 | 282 | 4.85E-40 | 120.99 | 0.4373 | 0.0333 | down |
| P27348 | 1433T | 14-3-3 protein theta | NLLSVAYKNVVGGR | 14 | 1 | 8 | 49 | 3.43E-11 | 105.17 | 0.4377 | 0.0174 | down |
| Q9GZZ1 | NAA50 | N-alpha-acetyltransferase 50 | FYKDVLEVGELAK | 13 | 1 | 3 | 37 | 5.27E-43 | 127.52 | 0.4401 | 0.0221 | down |
| O00422 | SAP18 | Histone deacetylase complex subunit SAP18 | ELTSLVKEVYPEAR | 14 | 1 | 7 | 74 | 3.68E-33 | 128.36 | 0.4409 | 0.0161 | down |
| P62333 | PRS10 | 26S proteasome regulatory subunit 10B | KIHIDLPNEQAR | 12 | 1 | 1 | 298 | 0.0004434 | 93.551 | 0.4425 | 0.0335 | down |
| P27708 | PYR1 | CAD protein | LSSFVTKGYR | 10 | 1 | 7 | 1411 | 8.42E-05 | 86.953 | 0.4477 | 0.0245 | down |
| Q9H9T3 | ELP3 | Elongator complex protein 3 | NLHDALSGHTSNNIYEAVKYSER | 23 | 1 | 19 | 206 | 5.29E-39 | 86.727 | 0.4485 | 0.0025 | down |
| P21127 | CD11B | Cyclin-dependent kinase 11B | DLKTSNLLLSHAGILK | 16 | 1 | 3 | 564 | 7.51E-07 | 70.889 | 0.4494 | 0.0446 | down |
| P29590 | PML | Protein PML | LQDLSSCITQGKDAAVSK | 18 | 1 | 12 | 394 | 1.27E-160 | 170.11 | 0.4561 | 0.0084 | down |
| P06493 | CDK1 | Cyclin-dependent kinase 1 | DLKPQNLLIDDKGTIK | 16 | 1 | 12 | 139 | 2.16E-34 | 122.97 | 0.4562 | 0.0013 | down |
| Q6ZNJ1 | NBEL2 | Neurobeachin-like protein 2 | KYGVQFILDALR | 12 | 1 | 1 | 1059 | 9.50E-05 | 68.069 | 0.4563 | 0.0108 | down |
| Q96JC1 | VPS39 | Vam6/Vps39-like protein | ELFPTGKQLEPLVAPLADGK | 20 | 1 | 7 | 204 | 5.95E-05 | 54.805 | 0.4568 | 0.0481 | down |
| Q13151 | ROA0 | Heterogeneous nuclear ribonucleoprotein A0 | LFVGGLKGDVAEGDLIEHFSQFGTVEK | 27 | 1 | 7 | 106 | 1.29E-56 | 92.95 | 0.4586 | 0.0164 | down |
| P21796 | VDAC1 | Voltage-dependent anion-selective channel protein 1 | NVNAGGHKLGLGLEFQA | 17 | 1 | 8 | 274 | 0 | 234.39 | 0.4599 | 0.0366 | down |
| Q8NI27 | THOC2 | THO complex subunit 2 | ATGFDGGNKADQLDYENFR | 19 | 1 | 9 | 1084 | 2.83E-23 | 84.559 | 0.4642 | 0.005 | down |
| Q9NRD8 | DUOX2 | Dual oxidase 2 | SHPQGLGPPAPEAPELGGPGLKK | 23 | 1 | 23 | 997 | 2.20E-44 | 92.474 | 0.4647 | 0.0301 | down |
| P12270 | TPR | Nucleoprotein TPR | LEKELENANDLLSATK | 16 | 1 | 3 | 351 | 0.0010579 | 50.284 | 0.4668 | 0.0073 | down |
| Q8WVV4 | POF1B | Protein POF1B | GSHFFPGNNVIYEKTIR | 17 | 1 | 14 | 168 | 2.50E-140 | 162.93 | 0.4669 | 0.018 | down |
| P21796 | VDAC1 | Voltage-dependent anion-selective channel protein 1 | AVPPTYADLGKSAR | 14 | 1 | 11 | 12 | 0.0001233 | 63.283 | 0.4686 | 0.0289 | down |
| Q99613 | EIF3C | Eukaryotic translation initiation factor 3 subunit C | TEPTAQQNLALQLAEKLGSLVENNER | 26 | 1 | 16 | 862 | 1.65E-190 | 157.94 | 0.4696 | 0.0402 | down |
| P06493 | CDK1 | Cyclin-dependent kinase 1 | GTIKLADFGLAR | 12 | 1;2 | 4 | 143 | 0.0011041 | 57.598 | 0.4718 | 0.0314 | down |
| Q5W0Z9 | ZDH20 | Palmitoyltransferase ZDHHC20 | ALPIYTTSASKTIR | 14 | 1 | 11 | 123 | 3.49E-08 | 101.11 | 0.4721 | 0.0017 | down |
| P63000 | RAC1 | Ras-related C3 botulinum toxin substrate 1 | HHCPNTPIILVGTKLDLR | 18 | 1 | 14 | 116 | 4.27E-27 | 112.24 | 0.4771 | 0.0164 | down |
| Q13228 | SBP1 | Methanethiol oxidase | TKLVLPSLISSR | 12 | 1 | 2 | 93 | 1.16E-05 | 83.045 | 0.486 | 0.0398 | down |
| Q75N03 | HAKAI | E3 ubiquitin-protein ligase Hakai | YDCKGGELFANQR | 13 | 1 | 4 | 74 | 2.60E-06 | 99.752 | 0.4862 | 0.0025 | down |
| P14618 | KPYM | Pyruvate kinase PKM | TATESFASDPILYRPVAVALDTKGPEIR | 28 | 1 | 23 | 115 | 3.93E-86 | 127.87 | 0.4887 | 0.0129 | down |
| P78527 | PRKDC | DNA-dependent protein kinase catalytic subunit | KGGSWIQEINVAEK | 14 | 1 | 1 | 4023 | 0 | 213.34 | 0.4893 | 0.01 | down |
| P07814 | SYEP | Bifunctional glutamate/proline--tRNA ligase | INEAVECLLSLKAQYK | 16 | 1 | 12 | 861 | 7.71E-12 | 94.728 | 0.4902 | 0.0469 | down |
| P02794 | FRIH | Ferritin heavy chain | AIKELGDHVTNLR | 13 | 1 | 3 | 147 | 2.29E-18 | 116.54 | 0.4912 | 0.0302 | down |
| Q92508 | PIEZ1 | Piezo-type mechanosensitive ion channel component 1 | LDLKDIPNAIR | 11 | 1 | 4 | 113 | 2.58E-06 | 105.57 | 0.492 | 0.0162 | down |
| Q9NXE4 | NSMA3 | Sphingomyelin phosphodiesterase 4 | KQLPDCIVGEDGLILTPLGR | 20 | 1 | 1 | 693 | 3.68E-13 | 72.184 | 0.4933 | 0.0185 | down |
| P62750 | RL23A | 60S ribosomal protein L23a | KLYDIDVAK | 9 | 1 | 1 | 115 | 0.0020757 | 112.17 | 0.4943 | 0.0492 | down |
| Q99973 | TEP1 | Telomerase protein component 1 | EELALYGKR | 9 | 1 | 8 | 1339 | 0.0382718 | 58.981 | 0.4995 | 0.0357 | down |
